# Positive cyberpsychology as a field of study of the well-being of people interacting with and *via* technology

**DOI:** 10.3389/fpsyg.2023.1053482

**Published:** 2023-02-23

**Authors:** Paweł Fortuna

**Affiliations:** Department of Experimental Psychology, The John Paul II Catholic University of Lublin, Lublin, Poland

**Keywords:** positive cyberpsychology, cyberpsychology, human-computer interaction, positive psychology, well-being, flourishing

## Abstract

The aim of the article is to postulate introducing and developing positive cyberpsychology (PCyb) as a subdiscipline of cyberpsychology, which emerges at the intersection of cyberpsychology, positive psychology, and well-being informed design, and focuses on studying determinants of human well-being through interactions with and *via* technology. The article presents the rationale for considering the emergence of PCyb based on the importance of research on the positive transformation of people in the era of progressive digitalization and cyborgization, and the growing partnership of cyberpsychology, positive psychology, and well-being informed design in the form of paradigms and ongoing research. Moreover, it highlights the need to reframe cyberpsychology dominated by the study of the “dark side” of technology and the need to integrate and increase the “visibility” of research results on the beneficial effects of technology. The article also accentuates the opening perspective of a more in-depth analysis of the positive transformation process than the one existing within the well-being informed design and underlines a broader plan of innovation use than is taken into account in cyberpsychology and positive psychology. Lastly, it discusses the use of the results of research conducted within PCyb in the design of new technologies, consulting, and education, as well as the possibility of strengthening the voice of psychologists in the debate about the future of humans functioning in the constantly changing technosphere.

## 1. Introduction

The pursuit of well-being (optimal functioning, flourishing) is inscribed in human nature, and reflection on happiness has accompanied people from antiquity (felicitology; Aristotle, 2012) through the beginnings of psychology (James, 1902), until now, when the study of its determinants has reached momentum with the development of positive psychology (Seligman, 1998). The rapid increase in innovation makes modern humans look for their own way to achieve and maintain well-being by experimenting with technology (referred to as STARA: Smart Technology, Artificial Intelligence, Robotics, and Algorithms; Brougham and Haar, 2018). In order to maximize the experience of well-being, they need innovations and online environments designed not only for their satisfaction and commitment, but also for nourishing mental strengths such as creativity, bravery, self-control, and humility. They need an understanding of the processes underlying the technology-supported positive transformation, as well as the competence to exert appropriate pressure on the market, and wisely use the acquired innovations. For these reasons, modern humans need strong support from the world of science, specifically from the area specialized in studying conditions and processes that contribute to well-being of people interacting with technology. The PCyb concept was developed independently by Burke (2021) and Fortuna (2021). The following focuses on the reasons for introducing PCyb, and a comparison of these approaches will be presented in a separate article (Burke and Fortuna, in preparation).^1^

PCyb appears as an approach that emerges at the junction of cyberpsychology (how technological innovations affect and depend on human behavior; Harley et al., 2018), positive psychology (what makes life most worth living; Peterson, 2008), and well-being informed design (how can technology be designed to support wellbeing or true flourishing; Peters et al., 2018). Focusing, like a lens, the interests of the above-mentioned areas, it centers on searching for an answer to the question, which broadly takes the form of a question: What conditions and processes contribute to the well-being achieved by people interacting with and *via* technology? As a result, it can explain the psychological determinants of the impact of technology on mental states, behaviors and interactions that promote well-being, determine the optimal usage of technology, and it can provide guidance on the specific features of innovations that favor beneficial interaction with them.

The above-mentioned issues are the subject of reflection of scientists, just like before the emergence of positive psychology, the issue of a good quality life was the subject of research (e.g., Diener et al., 1985; Csíkszentmihályi, 1990). It would be unnecessary to consider the idea of PCyb if the conducted research on the determinants of beneficial human-technology interactions had the character of systematic analysis, providing opportunity for a comprehensive approach to this phenomenon. There is a noticeable focus on the “dark side” of technology use in the cyberpsychology field (see Ancis, 2020), research carried out within positive psychology generally ignores the aspect of designing innovations (e.g., Prasetyo et al., 2022), and well-being informed, design related analyses have no ambition to explain the mental processes underlying the positive transformation of innovation users (Calvo and Peters, 2017).

PCyb, developing as a distinct subdiscipline of cyberpsychology, can primarily contribute to giving it a more balanced profile. If cyberpsychology is to provide a holistic picture of how the users of innovation function, it must reframe both, the “light” and “dark side” of technology use, through a balanced analysis. An opportunity for this is the development of models that manifest the partnership between cyberpsychology and positive psychology (e.g., Riva et al., 2012). However, positive psychology should not be treated as a “catalogue” of conceptual research frameworks. It is an area of lively discussion concerning the psychological mechanisms on which the shaping and enhancing mental strengths depends. In turn, intellectual cooperation with the well-being informed design gives an opportunity to take a broad look at the user experience process and systematically study its individual stages, from adoption of technology to the increase in well-being at the societal level. Compared to a well-being informed design, PCyb appears as a perspective that goes beyond the question of “how” to the question of “why” in explaining the positive impact of human-technology interaction. Attempts at deeper reflection on the conditions of positive transformation of users are already being made (Riva et al., 2016, 2018), but they require expansion and intensive research. The sign of PCyb’s presence in the world of science should be the constant integration and systematization of the fragmentary and dispersed research results on beneficial human-technology interactions. It would also be beneficial to increase the interest of academics in the “light side” of technology use, resulting in increased research work, adequate to growth of innovations and challenges faced by their users.

## 2. Reasons for introducing positive cyberpsychology

### 2.1. The well-being of people interacting with technology as a momentous research challenge

Advances in technology and the ways it is used have made the distinction between “the figure” and its “background” gradually blurred. People interacting with technological artifacts create hybrid systems (Loh and Loh, 2017), and the degree of fusion with artificial units can be placed on the cyborgization continuum (Jupiter, 2016): from interaction with static (PC), mobile (smartphone) and wearable (smart-glasses) technologies, to augmentation (connecting artifacts with human nervous system) and predicted transcendence (uploading data from, and downloading to, brains). Transhumanists see this fusion as a source of technological empowerment that offers a chance for super-prosperity (Pearce, 2007; Bostrom, 2014). Advanced work on cyborgic improvements is carried out in the military sector (Emanuel et al., 2019), and many actions are implemented as biohacking projects (Łukaszewicz-Alcaraz, 2020). Close cooperation of natural human beings, computers, cyborgs and bioroids is expected in business organizations, with the rational agent being able to perform roles that have so far been reserved only for people (e.g., supervisor or even CEO; Gladden et al., 2022). At the same time, the authors of the European Commission’s report defining “industry 5.0” (Industry 5.0, 2021), anticipate the deepening of human-computer interaction and postulate raising it to a higher level by focusing on human needs. Finally, considerations regarding superhuman AI lead to the conclusion that if we want advanced systems to support our needs and aspirations, we should clarify them first, because if we give control to machines that do not share them, we risk undesirable outcomes (Tegmark, 2017).

Progressive digitization and cyborgization is a challenge for researchers who can and should tangibly influence shaping innovations that, by enhancing the well-being of its users, can earn the term “positive” or “well.” The proof of their beneficial impact should be the empirically verified positive transformation leading to well-being of both individuals and wider systems, understood as “a state of happiness and contentment, with low levels of distress, overall good physical and mental health and outlook, or good quality of life”.^2^ Originally, positive subjective experience (hedonistic aspect; Fredrickson, 2001), individual characteristics (eudaimonistic aspect; Peterson and Seligman, 2004), and institutions and communities (social aspect; Kern et al., 2020) were considered three “pillars” of a good life. Later, this increased to five: positive emotions, engagement, relationships, meaning and accomplishment (PERMA; Seligman, 2011). The well-being determinant factors, which are the focus of positive psychology, also constitute a set of dependent variables under PCyb. Unlike positive psychology, these variables should be operationalized considering the technological context of their achievement. For example, the self-determination theory–based measure (Sheldon et al., 1996), widely used in positive psychology, has its counterpart in human-technology interaction studies, which has been used to assess the experience of need satisfaction and user experience in video game contexts (PENS; Ryan et al., 2006), and its modified version for non-game technologies (TENS; Peters et al., 2018). The richness of technology motivates the development of methods to measure positive user experiences initiated at the level of its adoption and contact with the interface, and then refer them to the results obtained through the use of tools recognized in positive psychology (e.g., PERMA Profiler; Butler and Kern, 2016).

### 2.2. The prospect of developing a new subdiscipline within cyberpsychology

Cyberpsychology, which historically emerged from the psychology of media and communication, is just grounding its position in the world of science, but the observation of the dynamics of its development leads to the conclusion that it is a mature area for the emergence of subdisciplines. The range of interests of cyberpsychology is very broad (Attrill, 2015; Connolly et al., 2016). It includes disciplines such as Human-Computer Interaction (although this connection needs to be specified), computer science, and engineering (Ancis, 2020), and its interdisciplinary and transgressive character is marked in connection with many paradigms grounded in psychology. While considering the future of cyberpsychology, Ogonowska (2021) lists six paradigms that profile the problems, research strategies and possibilities of applying the obtained results: psychodynamic, bio-medical, behavioral, cognitive, humanistic, and anthropological-cultural. Strong relationship of cyberpsychology with fields developed within psychology, and beyond, affects its thematic breadth and methodological richness. For example, within the psychodynamic paradigm, cyberpsychology relates to clinical psychology focusing on the study of neuroticism and problematic Internet activities (Marciano et al., 2020), and to media studies facilitating the study of attitudes toward computerized psychotherapy (McDonnell et al., 2014).

PCyb emerges from the humanistic paradigm focused on studying human development, from which positive psychology grew (Rogers and Maslow are referred to as “grandparents” of positive psychology; Duckworth et al., 2005), and the behavioral paradigm, focused on studying the impact of the environment on the activity of the individual, which in turn is associated with well-being informed design. We have noticed the partnership of cyberpsychology, positive psychology, and well-being informed design for a decade, which is reflected in the approaches that can be considered the “intellectual founding capital” of PCyb. The link between cyberpsychology and positive psychology is noticeable in Positive Technology, which is “the scientific and applied approach to the use of technology for improving the quality of our personal experience through its structuring, augmentation, and/or replacement.” (Riva et al., 2012, p. 69). In turn, the relationship between positive psychology and well-being informed design is evident in Experience Design (Hassenzahl, 2010) – an approach to shaping positive individual experiences through interactive products; Positive Computing (Snyder et al., 2021) – the study and development of technology designed to support people’s psychological flourishing; Positive Computing (Calvo and Peters, 2012) – a methodological framework for designing wellbeing-informed technology; Positive Design introduced “to take a step toward operationalizing the holistic phenomenon of human happiness in user-centered design processes.” (Desmet and Pohlmeyer, 2013, p. 16). It is also captured in the model Motivation, Engagement and Thriving in User Experience (METUX), which was developed “to form actionable insights with respect to how technology designs support or undermine basic psychological needs, thereby increasing motivation and engagement, and ultimately, improving user wellbeing.” (Peters et al., 2018, p. 1). It is worth emphasizing that these approaches still require empirical verification.

Another indicator of the discussed partnership is an increase in the number of studies on beneficial human-technology interactions. They cover: (a) positive influence: e.g., reduction of symptoms of depression and stress (Villani et al., 2016), resilience (Phippen and Street, 2022), self-control (Greaney, 2016), cyberactivism (Stiff, 2019), social support (Coulson and Buchanan, 2022), inclusion (Chadwick and Wesson, 2016), love and close relationships (Hamilton, 2016); (b) technology applications: e.g., e-learning (Lawn et al., 2017), digital therapy (Gaggioli and Riva, 2014), health management (Sillence and Briggs, 2018); (c) innovation: e.g., AI (Tomašev et al., 2020); videogames (Liang et al., 2014), virtual reality (Chirico et al., 2016), wearable devices (Patel et al., 2015) and (d) beneficial usage of technology (e.g., Ko and Kuo, 2009; Nguyen, 2021). This research is complemented by reports of active participation of psychologists in project teams (e.g., CHI, 2022) and popular science publications (Fortuna, 2021).

### 2.3. The need for reframing cyberpsychology

The “visibility” of approaches explaining and postulating beneficial effects of technology use, and related research, is surprisingly low. Analysis of the most relevant research papers related to cyberpsychology published in 2012–2019 revealed that most of the research was conducted in medicine, public health, and psychiatry (Singh and Singh, 2019). The thematic areas identified are: e-therapy, digital addiction, technostress, cyborgization, cyber therapy, and cyberbullying. Only 1.33% (*N* = 4) of the pool of analyzed texts showed a clear relationship between cyberpsychology and positive psychology. Similar observations are provided by the research review carried out by Ancis (2020). It revealed that the dominant areas of research are online criminal activity, negative impact of smartphone use, internet addiction, and impact of violent games on behavior. Reports of beneficial human-technology interactions do not affect the asymmetry of research predominance of pathological use of technology. It is significant that approaches linking positive psychology with cyberpsychology, and well-being informed design were not raised in the studies mentioned. These concepts are also omitted in The Oxford Handbook of Cyberpsychology (Attrill-Smith et al., 2019), and their authors do not author any of the chapters. One can get an impression that reports on the harmful effects of human-technology cooperation have a higher status than findings indicating the opposite.

Cyberpsychology focusing on the “dark side” of technology is like a ship tilted to one side. The development of PCyb should be conducive to achieving the desired balance, manifested in a more complete picture of human functioning in a technological environment. It is worth noting that the intention of introducing the PCyb idea is not to depreciate research aimed at revealing the negative dimension of technology use. Problematic user activities require clarification, but the dominant focus on them brings cyberpsychology dangerously close to a kind of “victimology.” An unintended side effect may be a perception of innovation use as something reprehensible (e.g., the third-person effect of the Internet has been found; Błachnio et al., 2010) or even technophobia (Khasawneh, 2018). Moreover, the popularity of innovation cannot be fully explained by succumbing to the influence of marketing or addiction, leading to compulsive behavior. Operating in hybrid systems is a must, so we need a balanced approach to the use of digital technology supported by the pillars of balanced cyberpsychology.

The first tasks carried out under PCyb should be to integrate and increase the “visibility” of dispersed research on the “bright side” of technology, as well as to make an effort to reinterpret the results of research conducted within cyberpsychology in the spirit of positive psychology. Combining the achievements of cyberpsychology, positive psychology and well-being informed design, and referring to holistic concepts of human well-being (e.g., PERMA; Seligman, 2011) will facilitate the view of the beneficial use of innovations from a broad perspective. In addition, as a point of reference, it will enable a critical assessment of the results of research highlighting the “dark” side of technology, showing human functioning fragmentarily, in relation to specific activities (Ancis, 2020). The main source of PCyb’s influence in creating a balanced image of cyberpsychology, however, should be the initiation of new research aimed at a more in-depth understanding of the processes underlying a positive transformation of users at various stages of interaction with technology.

### 2.4. Comprehensive research program focused on a positive transformation of a technology user

Positive changes in life are most often related to the implementation of a long-term action plan because interacting with technology is a complex process accompanied by a diverse spectrum of personal experiences. Therefore, in order to deepen knowledge about the processes underlying positive transformation, optimal ways of using innovations and improving them, it is necessary to adopt a broad framework of analysis. We find the appropriate inspiration in the METUX concept, which stems from well-being informed design (Peters et al., 2018). It lists six separable spheres of experience that can be influenced by technology design with desirable outcomes: (1) adoption (e.g., purchase), (2) interface (engagement with technology, satisfaction), (3) task (engagement with task, satisfaction), (4) behavior (experience of well-being during behavior), (5) life (increased life satisfaction), and (6) society (increased measures of societal well-being). The basis of METUX is self-determination theory (Ryan and Deci, 2000), but the mentioned spheres of user experience can be treated as a universal framework for the analysis of other well-being determinant factors studied in positive psychology (e.g., listed in the Positive Technology paradigm; Riva et al., 2012).

PCyb can also contribute to deepening the analysis conducted in the area of well-being informed design by verifying the hypotheses that are formulated within positive psychology, outside the context of technology. For example, the lively discussion on appreciation of beauty (Diessner et al., 2018), and its relationship to aesthetic experience (Skov and Nadal, 2020) and flow (Wanzer et al., 2020), require empirical verification. New light on the process of arousing and maintaining admiration for beauty can be shed using innovative solutions in immersive technologies (e.g., simulating imaginative processes in the artist’s mind) coupled with eye-tracking and neuroimaging techniques (e.g., Ansado et al., 2021; Chiquet et al., 2021). The possibility of influencing positive experiences through technology opens research perspectives similar to laboratory studies conducted in cognitive psychology (Galotti, 2004). It is desirable due to the criticism of the research methodology in positive psychology, dominated by questionnaire methods (Tucholska and Gulla, 2007).

Distinguishing the domains of user experience will specify the empirical analysis of the positive user transformation process. Consequently, the determination of more general regularities of human-technology interaction should be easier. Generalization of the obtained results can be based on suggestions taken from the recently introduced Systems Informed Positive Psychology (SIPP; Kern et al., 2020): (1) Boundaries and perspectives – factors affecting the inclusion of individuals into a positive system, e.g., technology acceptance and adoption (Modliński et al., 2023), Anthropocentrism (Fortuna et al., 2021); (2) Self-organization and interconnectedness – shaping favorable relationship patterns as a result of contact with the interface and the tasks performed, e.g., anthropomorphization of artifacts (e.g., Naneva et al., 2020); (3) Dynamics – study on maintaining motivation to interact with artifacts in different environments: e.g. engaging in a behavior that a technology is intended to support (e.g., Fryer and Bovee, 2016); (4) Adaptation and emergence – optimal functioning in the hybrid system and implementation of positive transformation effects: e.g., increasing wellbeing in workplace (e.g., Adhyaru and Kemp, 2022), increasing societal optimal functioning postulated by SIPP (Kern et al., 2020). The use of the above research key facilitates a link between areas integrated within PCyb and should be conducive to a comprehensive explanation of the effectiveness of technologies designed to improve well-being.

### 2.5. Development of “positive technology”

Many innovations are designed with the intention of maximizing positive impact on users (e.g., social robots created thanks to developmental cybernetics and robotics; Marchetti et al., 2018). The improvement in quality of life, expected by constructors and promised by marketers, requires constant verification and determination whether innovations deserve the “positive” or “well” attribute. Inspiring, in this context, is the distinction of hedonic, eudaimonic and social/interpersonal technologies introduced by the authors of Positive Technology (Riva et al., 2012). PCyb should revive the discussion on this classification because it is based on early assumptions about the pillars of positive psychology that have been extended from three to five (PERMA; Seligman, 2011). Its future modifications should be inspired by the concepts developed within positive psychology.

It should be emphasized that PCyb is not a proposal to promote the beneficial impact of technology on humans, providing arguments for digital utopians or leading to an increase in the number of techno enthusiasts. In the discussed context, PCyb appears rather as a source of critical voice toward the ethics of the design. Thanks to its close relationship with positive psychology, PCyb indicates that the quality of life of a person involved in technology is not only influenced by satisfaction and high assessment of the product usefulness, but also a whole package of variables related to mental strength and nature of the virtues (The Encyclopedia of Positive Psychology describes nearly 70 positive human experiences; Lopez, 2009). Equating well-being with the hedonistic aspect of a good life is too short-sighted, giving the illusion of no negative side effects. The desired impact of PCyb on the environment of a design should therefore be to “switch lights to high beam” to facilitate the determination of the conditions that should be met to maximize beneficial, sustainable effects of use and minimize undesirable effects. Noble postulates of ethical use of technology (see High-Level Expert Group on Artificial Intelligence, 2019; Industry 5.0, 2021) will not suffice and require support in the form of specific suggestions at the operational level, which stem from a holistic and practice-rooted view of the psychological determinants of human-technology interaction. Just as positive psychology contributed to the emergence of positive coaching (Trzebińska, 2008), PCyb can inspire the emergence of experts specialized in designing hybrid systems, thus supporting users who are currently independently seeking adequate ways to use artifacts with the belief that “everything will be fine.”

The effects of work carried out within PCyb should form an important source of pressure exerted on the innovation market by educating conscious consumers. The main source of knowledge about new technologies are pop culture narratives and information obtained from other users (Cave et al., 2018; Maison, 2019), which results in poor orientation in the area of applied innovations. Research on AI perception shows that there are profound differences between the identification of AI-driven systems between laymen and professionals (e.g., for laymen, a typical example of AI is a social robot, and an atypical one is a formula recommending products in an online store; Fortuna and Gorbaniuk, 2022). The issues offered for discussion in schools relate mainly to the “dark side” of technology, which is not surprising given the current image of cyberpsychology. Those include: cyberbullying, cyber-harrasment, trolling, sexting, pornography revenge, mysogynistic online communication, phishing, hate speech, cyberchondria (Taylor et al., 2017). One can hope that the idea of “humanics” (Aoun, 2018) will be implanted in education, which argues that, in addition to literacy and numeracy skills, a person should improve data literacy (data comprehension and analysis), technological literacy (understanding the way technology works) and human literacy (interpersonal communication and design). PCyb can significantly support the implementation of the idea of “humanics,” and before that occurs, it can extend the content of the curriculum to the well-being-enhancing use of technology.

## 3. Conclusion

PCyb currently has the status of an idea, an intellectual proposition that needs to be discussed. Naturally, a broad, insightful reflection on the positive relationship between humans and technology is more important than the emergence of the subdiscipline from cyberpsychology. In the article, I tried to demonstrate that the implementation of this goal will be easier as a result of reframing cyberpsychology achieved through integration of dispersed approaches and research. The noticeable, victimological aspect of research conducted within cyberpsychology, inspires closer cooperation between scientists working in this field and positive psychology. In turn, a stronger partnership between positive psychology and well-being informed design within PCyb opens the prospect of deepening the analysis of psychological determinants of the positive transformation of users. The implementation of new research projects meets the challenges posed by the development of psychology, AI and transhumanism. The effects of this work should manifest themselves in the design of innovations conducive to well-being, as well as in counseling and education. Importantly, increasing the “visibility” of research carried out as part of PCyb should contribute to strengthening the status of psychologists and their voice in the wider debate that Tegmark (2017) called “the most important conversation of our time” (p. 22). Despite the avalanche of innovation, the debate focused on the issue of clarifying human aspirations, to which the development of technology should be subordinated, is still at an early stage. The development of PCyb should contribute to consolidating this debate and facilitate a constructive conversation about the future.

## Author contributions

The author confirms being the sole contributor of this work and has approved it for publication.

## Conflict of interest

The author declares that the research was conducted in the absence of any commercial or financial relationships that could be construed as a potential conflict of interest.

## Publisher’s note

All claims expressed in this article are solely those of the authors and do not necessarily represent those of their affiliated organizations, or those of the publisher, the editors and the reviewers. Any product that may be evaluated in this article, or claim that may be made by its manufacturer, is not guaranteed or endorsed by the publisher.

## Footnotes

^1^Burke, J., and Fortuna, P. (in preparation). What (and why) is positive cyberpsychology?

^2^ https://dictionary.apa.org
